# Socio-cognitive processes in mild-moderate depression

**DOI:** 10.3389/fpsyg.2025.1541725

**Published:** 2025-07-04

**Authors:** Prathima Alevoor Raghavendra, Shantala Hegde, Mariyamma Philip, K. Muralidharan

**Affiliations:** ^1^Clinical Neuropsychology Unit, and Music Cognition Laboratory, Department of Clinical Psychology, National Institute of Mental Health and Neurosciences (NIMHANS), Bengaluru, KA, India; ^2^Clinical Neuropsychology Unit, Department of Clinical Psychology, Music Cognition Laboratory, Wellcome Trust/DBT India Alliance CPH - Intermediate Fellow (IA/CPHI/17/1/503348), National Institute of Mental Health and Neuroscienes (NIMHANS), Bengaluru, India; ^3^Department of Biostatistics, NIMHANS, Bengaluru, KA, India; ^4^Department of Psychiatry, NIMHANS, Bengaluru, KA, India

**Keywords:** social cognition, theory of mind, attribution style, emotion perception, music emotion perception, depression

## Abstract

**Background:**

Social cognition (SC), the ability to interpret and respond to social situations appropriately, is essential for effective interpersonal functioning. Challenges in these areas are a core feature of depression. Evidence shows mixed findings regarding the extent and presence of these deficits in depression, especially in its milder forms. SC comprises key processes such as the theory of mind (ToM), attribution style, emotion, and social perception. In addition to exploring emotion perception (EP) ability through faces and vocal stimuli, music has recently emerged as a valuable tool in studying EP, given the effectiveness of music intervention in improving mood and overall emotional functioning in patients with depression.

**Aim:**

This study aimed to explore social cognition abilities in patients with mild–moderate major depressive disorder (MDD) and investigate the relationship between SC and neurocognition in depression.

**Methods:**

Nineteen patients diagnosed with mild–moderate MDD and eighteen age-, sex-, and education-matched healthy controls (HCs) (*n* = 18) were assessed using the Social Cognition Rating tools in the Indian Setting (SOCRATIS), the NIMHANS Emotion Perception Test (NEPT; assessing facial and prosodic domains), and the Music Emotion Perception Test (MEPT).

**Results:**

Patients with MDD showed significant deficits in first-order ToM (FOT) compared to HCs (*p* = 0.01). On the music emotion recognition test, the MDD group rated the intensity of positive emotions (e.g., happiness) significantly lower than the HC group (*p* = 0.007). However, no significant group differences were found in the accuracy of emotion identification across facial, prosodic, or musical stimuli. Correlational analyses revealed trends toward significant positive associations between attention and second-order ToM (SOT; *r* = 0.58, *p* < 0.01), as well as between the executive function (EF) index and EP (*r* = 0.60, *p* < 0.01), SOT (*r* = 0.56, *p* = 0.01), and social perception (*r* = 0.60, *p* < 0.01).

**Conclusion:**

Individuals with mild–moderate depression show reduced FOT ability and emotion scaling of positive emotions on music excerpts. A potential association exists between neurocognitive (attention and EFs) and SC measures.

## Introduction

Depression is one of the leading mental health concerns (James et al., 2018; Proudman et al., 2021), often accompanied by impairment in social and interpersonal functioning. These difficulties have been linked to deficits in social cognition (SC), a set of cognitive and affective processes that help us perceive, interpret, and respond appropriately to social information (Kupferberg and Hasler, 2023; Ladegaard et al., 2014; Porter-Vignola et al., 2022). SC plays a crucial role in effective communication, perspective-taking, and developing adaptive coping strategies (Kan et al., 2004).

SC impairments, including difficulties in emotion identification, decoding affective prosody, and inferring the mental states of others, are strongly associated with the psychopathological severity of depression (Knight and Baune, 2019; Porcelli et al., 2019). Misperception of social cues, perceived social rejection, and hypervigilance around social situations as a result of SC impairments can further lead to a sense of loneliness, social isolation, poor social support, interpersonal conflicts, and altered quality of life in individuals with depression (Kupferberg and Hasler, 2023; Saris et al., 2017; Weightman et al., 2019).

While SC has been extensively explored in conditions such as psychosis and autism, research in depression is relatively limited. The extent of SC impairment in depression is often subtle (Bazin et al., 2009; Wang et al., 2008; Weightman et al., 2019), and linked to illness severity (Air et al., 2015), yet their impact can be clinically significant. The systematic exploration of SC so far has been limited to individual processes [such as the theory of mind (ToM) and emotion recognition], with limited exploration of SC as a cohesive construct (Weightman et al., 2014).

### Key components of SC and its relation to depression

Broadly, SC encompasses key processes, including ToM, attribution style, emotion perception (EP), and social perception (Green, 2005; Etchepare and Prouteau, 2017; Pinkham et al., 2013). Of these, facial EP (FEP) and ToM are SC's most extensively studied facets (Sencan, 2019).

#### Theory of mind

ToM refers to the ability to interpret the complex mental states of others (Premack and Woodruff, 1978). It is generally conceptualized into two subsystems: *cognitive ToM* (interpretation of beliefs and intentions) and *affective ToM* (inferences regarding the emotional states of others). Studies have shown impaired ToM in depression, highlighting deficits in both cognitive and affective ToM tasks (Ladegaard et al., 2014; Wolkenstein et al., 2011; Zobel et al., 2010). The ability to interpret mental states involves both automatic, stimulus-driven processes and reflective, controlled processes, which can be disrupted in those with depression, leading to biases in interpreting others' intentions (Olsson and Ochsner, 2007). Not all evidence, however, shows ToM impairments, with some studies reporting ToM deficits to be either temporary (that improves during remission) (Ladegaard et al., 2014) or even comparable to healthy controls (HCs; Wilbertz et al., 2010).

#### Attribution style

Attribution refers to the cause that individuals ascribe to some event. Earlier cognitive models of depression proposed that individuals with depression have a depressogenic inferential style resulting in the attribution of adverse life events to internal, global, and stable causes (Abramson et al., 1989) and positive events to external, specific, and unstable causes (Seligman et al., 1979). These attributions can directly influence emotional responses, which may explain why individuals with depression show heightened sensitivity to negative social cues. Despite widespread acceptance of depressogenic attribution styles, research findings are inconsistent, particularly in mild depression (Alloy and Abramson, 1988; Dennard and Hokanson, 1986).

#### Emotion perception

EP, an individual's ability to recognize and understand others' emotional states is among the most consistently impaired SC processes in depression (Dalili et al., 2015; Kan et al., 2004; Kraus et al., 2019; Peron et al., 2011; Phillips et al., 2003). FEP tasks are the most commonly employed paradigms in depression, revealing poor EP accuracy (Dalili et al., 2015; Kohler et al., 2011; Krause et al., 2021; Naranjo et al., 2011; Schirmer and Adolphs, 2017), negative interpretation of neutral faces (Leppänen et al., 2004), and blunted responsiveness to positive emotions (Bourke et al., 2010; Joormann and Gotlib, 2006; Langenecker et al., 2005; Surguladze et al., 2005; Suslow et al., 2004). These observations align with cognitive theories of depression (Beck, 1963), highlighting mood-congruent attentional biases (Bourke et al., 2010; Bower, 1981). EP co-occurs across many modalities, such as speech prosody, body language, and gestures (Schirmer and Adolphs, 2017; Uekermann et al., 2008) and is not limited to facial expressions alone. Emotions are recognized quickly and accurately when presented in multimodal formats (a combination of face and voice) (Garrido-Vásquez et al., 2011; Paulmann et al., 2009; Schirmer and Adolphs, 2017). A few studies exploring EP through prosody in depression reported that negativity bias outlined for FEP could also be extended to vocal stimuli (Peron et al., 2011; Uekermann et al., 2008).

Interestingly, a growing body of evidence explores music emotion perception (MEP) as a novel dimension of EP (Al'tman et al., 2012; Naranjo et al., 2011; Punkanen et al., 2011).

#### Music emotion perception

Music is a powerful medium through which emotions are evoked and conveyed (Juslin and Laukka, 2004). Our ability to recognize or respond to music-evoked emotions is present from infancy (Trehub, 2003), strongly justifying musical stimuli in emotion studies. Music emotion studies have primarily explored EP (Juslin and Laukka, 2004; Punkanen et al., 2011), physiological changes induced by music (Hodges, 2011), and emotion regulation by music (Saarikallio, 2010). Individuals with depression have been shown to react more intensely to sad music (Bodner et al., 2007), which has been interpreted through the lens of cognitive theories of depression that highlight negative schemas and bias toward sad emotions (Bodner et al., 2007). MEP deficits, if present, may reflect broader impairments in perceptual-affective processing. Exploring EP deficits through music, in addition to faces and prosody, could give us valuable insights into whether they are due to a disruption in more fundamental perceptual attunement mechanisms, irrespective of modality, or whether contextual cognitive biases in interpersonal settings primarily drive them.

In summary, given limited studies with inconsistent findings in the area of SC in depression, it is important to have a deeper understanding of these abilities in order to gain further insights into the clinical condition. Although the majority of studies have included patients with severe depression, it is imperative to investigate whether the nature of these deficits is similar in mild–moderate depression (Hale, 1998; Kohler et al., 2011; Ladegaard et al., 2014; Peron et al., 2011; Van Vleet et al., 2019; Zobel et al., 2010). Additionally, examining EP deficits through music offers a novel lens to capture perceptual-affective disturbances, as music engages core emotional systems. This is also relevant, given the increasing evidence for the use of music therapy in ameliorating cognitive and emotional disturbances associated with various neurological and neuropsychiatric conditions, including depression, anxiety, and schizophrenia (Aalbers et al., 2017; Geretsegger et al., 2017; Hegde, 2014; Kraus et al., 2019; Thaut, 2010).

The present study aimed to examine SC in the domains of ToM, attribution style, and EP in individuals with mild–moderate depression compared to matched HCs. This sample's neurocognitive and music-cognitive abilities have been reported previously (Raghavendra et al., 2022). In this study, we have also examined the relationship between neurocognition and SC.

## Methodology

### Participants

Patients diagnosed with mild or moderate MDD (*n* = 19) and age-, sex-, and education-matched HCs (*n* = 18) comprised the sample. The sample size was calculated based on estimates from previous studies using 80% power and a 0.05 significance level (Naranjo et al., 2011). The diagnosis was made as per the 10th revision of the International Classification of Diseases (ICD-10; World Health Organization, 1993). Patients were recruited from the outpatient services of a tertiary mental health and neuroscience institute. The author MK carried out a clinical evaluation and confirmed the diagnosis. Patients diagnosed with severe MDD, bipolar depression, and suicidal ideation, and those having a current or past history of comorbid psychiatric, neurological, or neurodevelopmental conditions were excluded from the current study. HCs were recruited from the community using the convenient sampling method, where participants were recruited based on their ease of availability. All participants were right-handed, as screened on the Edinburgh Handedness Inventory (Oldfield, 1971).

### Tools

Sociodemographic and clinical details and music behavior data were recorded. The music behavior data included details such as participants' music listening habits, music preferences, attitudes toward using music in interventions, perceived benefits of music listening, and self-rated ability to perceive rhythm and remember tunes on a scale of 1–10, with higher scores corresponding to greater ability (Raghavendra et al., 2022).

### Screening tools

The Hamilton Depression Rating Scale (HAMD; Hamilton, 1986) was used to assess the severity of depression in patients. The Kessler Psychological Distress Scale (K6) was used to screen for psychological distress in HCs.

### Outcome measures

1. *NIMHANS Emotion Perception test (NEPT)* assessed EP across facial and prosody communication channels. The tool has been validated on the Indian population. It comprises six subtests of emotion perception: facial expression identification, prosody identification, facial expression discrimination, prosody discrimination, facial expression-prosody discrimination, and verbal-prosody discrimination. Facial emotion identification and discrimination tasks used still photographs of faces depicting varied emotions. Prosody identification and discrimination consisted of samples of spoken sentences in gibberish language or meaningful sentences (in verbal prosody identification/discrimination). In identification tasks, participants were asked to choose the emotion depicted through faces or voices from the list of seven emotions: happy, sad, anger, fear, surprise, disgust, and neutral. In discrimination tasks, the control tasks, participants were asked to discriminate whether the emotions portrayed in each pair were the same or different (Rani, 2009).
2. *The Musical Emotion Perception task (MEPT)* consisted of 32 musical excerpts (van Tricht et al., 2010). Each excerpt conveyed a specific emotion, and participants were asked to identify the emotion from a list of emotions—happy, sad, fearful, and angry. The participants were also asked to rate the intensity of emotion perceived in each excerpt, on a scale of 1–10, higher score indicating greater intensity.
3. *SC Rating tools in the Indian Setting (SOCRATIS)* assessed social cognitive abilities in three domains: (1) ToM task, which included first-order and second-order ToM tasks (FOT/SOT), Metaphor irony tasks, and faux pas recognition test, assessing both cognitive and affective ToM. (2) Attribution style—Internal, Personal, and Situational attributions questionnaire (IPSAQ; Kinderman and Bentall) was used to assess attribution style. This comprised 32 hypothetical social situations, and respondents had to make causal attributions, which were classified as internal (to self) or external (personal or situational). (3) Social Perception SoCuReTi (Social cue recognition test in an Indian setting)—four high emotion and four low emotion videos of social situations were shown to respondents. For each video, respondents answer true–false statements about social cues (rules, affect, and goals) and non-social facts (sights and sounds) (Mehta et al., 2011).

All the scores were computed automatically by the SOCRATIS software [SOCRATIS was developed at NIMHANS (National institute of mental health and neurosciences), Bangalore, India (Mehta et al., 2011)]. Scores were computed for *ToM* [FOT and SOT index, faux pas composite index (FPCI)], *attribution style* [externalizing bias (EB) and personalizing bias (PB)], and *SoCuReTi* [Social perception index (SPI)]. A positive EB score suggests that the individual attributes more positive events to self than adverse events (self-serving bias). As a corollary, it also indicates the tendency to attribute adverse events to external causes. On the other hand, A PB score above 0.5 indicates a tendency to use personal rather than situational external attributions for adverse events.

### Procedure

The current study received approval from the Institute's Ethics Committee (No. NIMH/DO/BEH.Sc.Div/2017-18). All the tools and scales were administered after obtaining written informed consent from the participant. The assessments were conducted in a controlled laboratory setting within a tertiary mental health care center, and the total assessment duration was ~2 h. After collecting relevant sociodemographic and clinical data, these tests were administered in the sequence listed in the tools section. The participant's responses for NEPT and MEPT were manually recorded and scored, whereas the SOCRATIS tool was administered through computerized software, automatically generating scores for each sub-test.

Upon completion, feedback about test findings was provided, and psychotherapy referrals were facilitated as needed.

### Statistical analysis

The raw data were coded and analyzed for Windows using IBM SPSS Statistics (Version 22.0; IBM Corp., 2013). Descriptive analysis, such as mean, standard deviation, and frequencies, was carried out for the sociodemographic and clinical data (Tables 1, 2).

**Table 1:** Sociodemographic details of participants (Raghavendra et al., 2022).

**Variables**	**Sub-categories**	**Scores**	**Group**		***t*/χ^2^**
			**MDD (*****N*** = **19)**	**HC (*****N*** = **18)**	
Age (in years)		Mean (SD)	28.0 (6.0)	28.16 (7.57)	0.07
Sex	Male	Frequency	7	7	0.01
		%	36.8	38.19	
	Female	Frequency	12	11	
		%	63.2	61.1	
Marital status	Married	Frequency	8	6	0.30
		%	42.1	33.3	
	Unmarried	Frequency	11	12	
		%	57.9	66.7	
Education (in years)		Mean (SD)	17.0 (2.13)	17.56 (2.13)	0.76
Employment	Unemployed	Frequency	10	8	0.24
		%	52.6	44.4	
	Employed	Frequency	9	10	
		%	47.4	55.6	

**Table 2:** Clinical status of the MDD group (*n* = 19) (Raghavendra et al., 2022).

**Variable**	**Response categories**	**Descriptive**	**Value**
Depression—severity	Mild	Frequency	06
		%	31.57
	Moderate	Frequency	13
		%	68.42
Duration	No. of years	Mean (SD)	3.51 (4.6)
Age of onset	In years	Mean (SD)	24.5 (7.15)
Scores on HAM-D	HAM-D total	Mean (SD)	14.2 (2.06)
Subjective cognitive complaints (on a scale of 1–10		Mean (SD)	6.10 (1.6)

HAM-D, Hamilton Depression Rating Scale.

The Mann–Whitney *U*-test, a non-parametric test, was used to compare the two independent groups—MDD and HC on tests of EP (facial, prosody, and music EP domains) and SC (Tables 3, 4). Effect sizes (rank biserial correlation) were calculated to understand the magnitude of differences. Spearman's correlation assessed the relationship among EP, SC, and neurocognitive indices (Table 5). These statistical approaches were chosen due to violations of normality assumptions and limited sample size (Howell, 2013; Kothari, 2004).

**Table 3:** Comparison between MDD and HC groups on domains of social cognition using Mann–Whitney *U*-test.

**Variable**	**Median (Q3–Q1)**		**Test statistic (*U*)**	***p*-value**	**Effect size**
	**Depression (*****n*** = **19)**	**Healthy controls (*****n*** = **18)**			
Facial emotion identification	12 (13–12)	11 (13–11)	113.5	0.07	0.33
Facial emotion discrimination	12 (13–11)	12.5 (13.2–11.7)	149.5	0.51	0.12
Prosody identification	11 (12–10)	10.5 (12–9)	153.0	0.58	0.10
Prosody discrimination	12 (13–9)	12 (13.25–11)	137	0.32	0.19
Facial expression-prosody discrimination (FE-PD)	11 (12–10)	11 (12.25–10)	149.5	0.51	0.12
Verbal-prosody discrimination (V-PD)	12 (13–12)	12 (13–10)	153.5	0.59	0.10
Overall emotion perception (as on NEPT)	70 (73–66)	69 (75–64)	0.09	0.92	0.01
Music emotion perception	21 (20.2 11.2)	22 (23–19.75)	165	0.78	0.03
FOT Index	1.0 (1.0–0.75)	1.0 (1–1)	117	0.01^*^	0.33
SOT Index	0.75 (1–0.75)	0.87 (1–0.75)	154.5	0.59	0.09
FPCl	0.97 (1.02–0.8)	1.0 (1.02–0.97)	140.5	0.35	0.18
EB	5.0 (9.0–2.0)	4.0 (6.5–0.75)	132.5	0.24	0.22
PB	0.6 (0.8–0.5)	0.50 (0.8–0.4)	129	0.20	0.24
SPI	0.92 (0.9– 0.8)	0.91 (0.96–0.89)	154	0.61	0.09

^*^Significant.

NEPT, NIMHANS emotion perception test; FOT, first-order theory of mind; SOT, second-order theory of mind; FPCI, faux pas composite index; EB, externalizing bias; PB, personalizing bias; SPI, social perception index.

**Table 4:** Comparison between MDD and HC groups on rating intensity of perceived emotions on music emotion perception test using the Mann–Whitney U-test.

**Variable**	**Median (Q3–Q1)**		***U*-value**	***p*-Value**	**Effect size**
	**DP (*****N*** = **19)**	**HC (*****N*** = **18)**			
Happiness	6.9 (7.8–5.5)	08 (9.07–6.7)	84.5	0.003^*^	0.51
Sadness	6.5 (7.5–5.6)	6.8 (8.02–5.77)	135	0.28	0.21
Fear	6.4 (7.0–5.75)	6.9 (8–6.05)	126	0.26	0.27
Anger	6.1 (7.4–5.4)	6.95 (8.45–6)	124.5	0.16	0.27

^*^Significant.

**Table 5:** Relationship between neurocognitive indices and social cognitive processes.

**Indices**		**Emotion perception**	**FOT index**	**SOT index**	**FPCI**	**EB**	**PB**	**SPI index**	**MER**
Focused attention	*r*	0.35	0.37	0.58^*^	0.338	−0.09	−0.11	0.25	0.08
	*p*	0.13	0.11	0.009	0.15	0.70	0.62	0.30	0.72
Executive function	*r*	0.60^*^	−0.21	0.56^*^	0.48	0.41	0.02	0.61^*^	0.19
	*p*	0.007	0.37	0.01	0.03	0.86	0.91	0.006	0.42
Learning and memory	*r*	0.47	0.13	0.39	0.17	−0.04	−0.14	0.114	0.40
	*p*	0.03	0.57	0.09	0.47	0.84	0.55	0.64	0.08

^*^Marginally significant (0.001 < p < 0.01).

FOT, first-order theory of mind; SOT, second-order theory of mind; FPCI, faux pas composite index; EB, externalizing bias; PB, personalizing bias; SPI, social perception index; MER, music emotion recognition.

Given the multiple correlation tests between variables carried out in the study, the significance threshold, alpha *(*α), was set at 0.001 to account for multiplicity using the formula (α/number of tests), that is, 0.05/32 (Streiner and Norman, 2011).

The correlations with *p*-values between 0.01 and 0.001 (0.01 > *p* > 0.001) showed a trend toward significance, indicating potential associations warranting further exploration.

## Results

The sample consisted of 19 patients diagnosed with mild–moderate MDD (MDD: *n* = 19; M:F = 7:12; mean age (years) 28±6), and age, sex, and education matched HCs [*n* = 18; M:F = 7:11; mean age (years) 28.1±7.5]. The two groups did not differ significantly on any of the sociodemographic variables (Table 1).

### Comparison of performance on tests of SC between MDD and HC participants

The groups were compared on all the key processes of SC, including ToM, attribution styles, social perception, and EP, including MEP (Table 3).

#### Theory of mind

The groups differed significantly on the FOT task, with a medium effect size (*p* = 0.01, effect size = 0.33), with the MDD group performing poorer than HC. However, there was no significant difference between the groups on the SOT and FPCI.

#### Attribution style

No significant difference was observed between the groups on both EB and PB. However, on comparing the median scores, it was observed that the MDD group had a greater tendency toward both PB and self-serving bias.

#### Social perception

The groups did not differ significantly on SPI.

#### Emotion perception

The groups did not show significant differences in the accuracy of perceiving emotions (emotion identification) on facial or prosody identification and discrimination (Table 3).

#### Music emotion perception

The findings indicate that the groups differed significantly on intensity rating for “happiness” emotion, with a large effect size, on the MEP (*p* < 0.001; effect size = 0.51; Table 4), although they did not differ on the accuracy of EP. The excerpts conveying “happiness” were rated lower in intensity by the MDD group in comparison to the HC group (Table 4).

### Correlational analyses

The relationship between neurocognition and SC domains was explored using Spearman's rho (Table 5). For neurocognitive data, the neurocognitive indices (attention, executive functions (EFs), and learning, and memory indices) were derived from the dataset of our previously published study on music and neurocognition in depression (Raghavendra et al., 2022).

Of all neurocognitive indices, there was a trend toward significance for the correlation between EF index and EP (*r* = 0.60, *p* = 0.006), SOT (*r* = 0.56, *p* = 0.01), and SPI (*r* = 0.61, *p* = 0.007). Focused attention was also marginally associated with the SOT (*r* = 0.58, *p* < 0.01).

No significant correlations were seen between (1) learning and memory index and any of the SC variables, (2) attribution (PB and EB) and any of the neurocognitive indices, and (3) FPCI and any of the neurocognitive indices.

## Discussion

The present study explored SC in individuals with mild–moderate depression (MDD) in domains of ToM, attribution style, and EP (facial, prosodic, and musical), in comparison to age, sex, and education-matched HCs.

Individuals with MDD showed significantly poorer FOT when compared to HC, which is partially consistent with the literature suggesting ToM deficits in depression (Bertoux et al., 2012; Bora and Berk, 2016; Kettle et al., 2008; Wilbertz et al., 2010). However, performance on SOT and faux pas recognition tasks did not show group differences, indicating preserved ability. The research exploring ToM abilities in depression generally has shown mixed findings, with few studies indicating no consistent underperformance on ToM tasks (Nestor et al., 2022). Some studies similarly showed preserved cognitive ToM (Berecz et al., 2016), but deficits in affective ToM (Harkness et al., 2011; Lee et al., 2005; Nejati et al., 2012). In our study, FOT was impaired, whereas SOT, which is more cognitively complex, was preserved. We speculate that difficulties in the initial attention allocation, possibly due to subtle cognitive deficits (Raghavendra et al., 2022) or affective/motivational fluctuations, may have caused this. Though higher-order ToM appears preserved, its clinical relevance is crucial. Tasks requiring higher-order reasoning abilities may still challenge these patients in day-to-day situations. Using dynamic and ecologically valid measures may help assess subtle deficits more effectively.

Significant group differences were not observed even on attribution style, which contrasts classic attribution models suggesting a depressogenic inferential style (Abramson et al., 1978, 1989). Our findings may be influenced by milder depression symptom severity, which is associated with enhanced attribution accuracy (Alloy and Abramson, 1988). Although the groups did not differ significantly in attribution styles, the MDD group showed tendencies toward self-serving bias (positive scores on EB) and personalizing bias (attributing adverse events to people rather than situations). These patterns align with previous reviews suggesting that, while individuals with depression may agree more with internal attributions for failure, they tend to favor external attributions for failure and internal attributions for success when compared to those without depression (Coyne and Gotlib, 1983; Zuroff, 1981).

Additionally, the groups did not differ in the accuracy of EPs via faces, prosody, or music. These findings align with previous studies reporting intact FEP abilities in depression (Kan et al., 2004; Peron et al., 2011; Schaefer et al., 2010; Suslow et al., 2004), although certain meta-analytic studies suggest mixed results with small-to-moderate effect sizes on facial emotion recognition and prosody identification capacity (Dalili et al., 2015; Kohler et al., 2011; Krause et al., 2021; Tang et al., 2020). However, the MDD group differed in rating the intensity of positive emotion (happiness) expressed by the excerpt, which can be attributed to blunted reactivity or reduced reward sensitivity (Pizzagalli et al., 2008). This dissociation between accurate EP and reduced intensity rating highlights the difference between perceived emotion (cognitive appraisal) and felt emotion (nuanced affective experience) (Kallinen and Rkanavaja, 2006). Few studies that have explored emotions induced by music in those with depression (Bodner et al., 2007; Sakka and Juslin, 2017) showed some differences in the way individuals with depression respond to music. Overall, the EP accuracy seems to be intact, with alterations seen in interpretations of emotional intensity. It is important to note, however, that the EP task used in the current study captured the ability to perceive universal basic emotions and did not include complex emotions such as pride, shame, or guilt. This could have resulted in the ceiling effect, masking subtle deficits in milder forms of depression.

Overall, SC deficits in those with mild–moderate depression appear subtle. Symptom severity can, however, moderate these deficits (Air et al., 2015), with evidence suggesting that mild depressive symptoms can paradoxically enhance SC performance (Harkness et al., 2005) due to enhanced sensitivity to social cues as a compensatory mechanism in the symptomatic phase. This could explain the relatively preserved SC in our sample, whereas severe depression can lead to generalized impairment due to motivational symptoms overshadowing social sensitivity (Lee et al., 2005). In addition to severity being a crucial factor, it is important to note that most participants were young adults and educated (Table 1) in our sample. These variables are also known to modulate SC measures (De Souza et al., 2018; Fittipaldi et al., 2024; Li et al., 2012).

### Relationship between SC and neurocognitive indices in depression

The current study explored the association between SC and neurocognition in MDD. Neurocognitive indices were derived from a prior study published by the same authors (Raghavendra et al., 2022). Correlational analysis revealed marginal associations between EFs with emotion perception, SOT, and social perception. Focused attention was also marginally associated with the SOT, though significant relationships were not found between any variables.

The current literature on depression highlights a significant relationship between neurocognition and SC abilities (Uekermann et al., 2008; Zobel et al., 2010). In particular, components of EFs are known to be implicated in SC (Yang et al., 2015). Understanding and interpreting socio-emotional, such as affective decoding, understanding social cues, others' mental states, and intentions, requires both attention and EFs. Specifically, attention to relevant details, working memory, cognitive flexibility, and inhibitory control helps individuals hold and manipulate the current information based on context and memories and switch to an alternative perspective in a social situation while suppressing one's perspective (Moreau and Champagne-Lavau, 2014; Pagnoni et al., 2022; Thoma et al., 2011; Yang et al., 2015).

For example, identifying emotion through faces requires active visual scanning of the stimulus and integration of the visual information with pre-existing semantic knowledge about facial expressions, requiring working memory and inhibitory control (Muñoz Ladrón de Guevara et al., 2021). Similarly, EF and affective prosody recognition are also related, especially when the affective and semantic content of the prosodic stimuli are incongruent. Specific SC–neurocognition associations in our study suggest that individuals with mild–moderate depression may rely on executive and attentional resources actively for specific SC tasks, but not uniformly across all SC measures.

The absence of significant associations between SC constructs and learning and memory index (LMI) warrants further exploration, particularly given the known role of our memories from the past (autobiographical memory) and childhood experiences in navigating social information (Spreng, 2013; Takim et al., 2024). Although memory aids in retrieving, updating, and interpreting information about people and situations, this relationship may not be captured by standard memory assessments. The memory tasks, like the ones used in this study, focus on real-time learning and retention of new information (word lists or a geometric figure) with limited personal relevance. On the other hand, the SC tasks engage real-time cognitive processes, such as EF, which may explain the stronger links between EF and SC in previous studies (Lancaster et al., 2003). Moreover, our sample showed minimal cognitive deficits (Raghavendra et al., 2022). In severe conditions, where both EF and memory are significantly impaired, SC deficits may become more pronounced (Lancaster et al., 2003).

Attribution style also showed no significant associations with neurocognition. Attribution style, which is generally trait-based and describes stable dispositions shaped by our personality and schemas (Peterson and Seligman, 1984), may have minimal loading on active cognitive processes.

Surprisingly, the faux pas index (a relevant component of ToM) did not correlate with EF, despite evidence supporting links between ToM and EFs (Förster et al., 2018; López-Navarro, 2018; Pagnoni et al., 2022; Schmid et al., 2021). While a few studies have examined faux pas recognition as an individual construct, to our knowledge, none have done so in depression. A recent study that explored these variables in individuals with alcohol use disorder reported that the overall faux pas recognition scores did not correlate with EF. However, specific components (e.g., faux pas knowledge and faux pas identification) related significantly to EF measures such as working memory, inhibition, and mental flexibility (Schmid et al., 2021). The findings also indicated that these variables may involve overlapping but distinct cognitive mechanisms, especially those with milder deficits. In our study, the use of composites did not allow us to explore domain-specific associations. Although correlations between FPCI and EF were not significant, especially after the correction (*p* = 0.03), the trend suggests a possible link worth exploring with a larger sample.

These interpretations cannot be overgeneralized and should be viewed with caution due to limitations such as small sample size and lack of robust analyses. In addition, illness severity could play a crucial role in defining the relationship between SC and neurocognitive variables, which is worth exploring in future studies. Further studies incorporating ecologically valid cognitive measures could capture the complexity of these interactions.

## Conclusion

The present study reveals that individuals with mild–moderate MDD show FOT deficits and differences in the subjective experience of emotional intensity, particularly for positive affect, compared to HC. A potential association exists between attention and SOT and EFs with emotion perception, SOT, and social perception.

This is one of the few studies exploring the elements of SC and its relation to neurocognition, especially in milder forms of depression. However, most studies have explored associations between severity and social cognitive measures in heterogeneous clinical samples of varying severity (Air et al., 2015; Gollan et al., 2010). A limited number of studies have examined EP in depression through a multimodal approach (Campanella et al., 2010; Garrido-Vásquez et al., 2011; Müller et al., 2013; Scibelli et al., 2016), with fewer addressing this topic in an impersonal domain like music (Naranjo et al., 2011). The comprehensive approach, encompassing various domains of SC, sets it apart from studies focusing on individual tests or domains. These findings have implications for adding more profound value to our understanding of these constructs, designing socio-cognitive interventions, and using music therapy in depression. Intervention designs aiming to improve SC in depression may need to be tailored separately through cognitive remediation approaches. Finally, the relative preservation of SC in mild–moderate depression indicates a window for early intervention before the emergence of global deficits, as seen in severe cases.

### Limitations and future directions

We acknowledge several limitations of this study. The small sample size challenges the power and the generalizability of the findings, and the cross-sectional design prevents causal inferences about the directionality between SC and neurocognition. Longitudinal and multi-site studies with larger sample sizes, the use of multivariate statistics to explore mediators and moderators, the inclusion of Indian music excerpts for cultural relevance, and the incorporation of dynamic and ecologically valid measures can enhance a more profound understanding and reliability of these findings. It is also crucial to explore individual EF measures in relation to specific SC measures, given evidence for possible domain-specific associations.

## Data Availability

The raw data supporting the conclusions of this article will be made available by the authors, without undue reservation.
